# Dental Education in the Time of COVID-19 Pandemic: Challenges and Recommendations

**DOI:** 10.3389/fmed.2021.648899

**Published:** 2021-05-31

**Authors:** Mohamed G. Hassan, Hala Amer

**Affiliations:** ^1^Department of Orthodontics, Faculty of Oral and Dental Medicine, South Valley University, Qena, Egypt; ^2^Department of Orthodontics, Faculty of Dentistry, October 6 University, Giza, Egypt; ^3^Department of Pediatric Dentistry and Dental Public Health, Faculty of Dentistry, Alexandria University, Alexandria, Egypt

**Keywords:** dental school, dental students, dental education, COVID-19, pandemic

## Abstract

Moving within the second wave of the coronavirus (COVID-19) pandemic, dental education delivery has been profoundly affected by this crisis, so has the structure, evaluation, and future of dental education. Both pre-clinical and clinical dental education have experienced challenges ranging from fully online educational content to limited dental training for senior dental students. This crisis appears to be a tipping point that produced confusion in dental teaching especially clinical sciences. Although medical institutions immediately started to adapt to the unexpected COVID-19 crisis, dental and oral health educational services are profoundly impaired due to the dental team's propinquity to the patient and the aerosols generated during routine dental therapeutic procedures. Dental students unlike other medical students are considered to be at the highest risk due to the nature of their clinical training that includes working in the oral cavity of patients using aerosol-generating equipment. Some dental schools have taken the leadership and documented their modifications during this pandemic; however, there is a serious need for further investigation and wide range screening of the situation in the dental schools during the COVID-19 crisis. The aim of this mini-review is to present these challenges and how academic dental institutions have implemented strategies to overcome them.

## Introduction

December 2019, Coronavirus disease 2019 (COVID-19) was firstly spotted in Wuhan, China, as pneumonia of unknown origin (1). COVID-19 flare-up spread rapidly not only in China, but also worldwide, therefore, on 11 March 2020, the World Health Organization (WHO) affirmed the SARS-CoV-2 virus, more commonly known as COVID-19 as a pandemic (2). The overall number of confirmed cases and mortalities are 78,194,105 and 1,754,369, respectively, in 216 countries as of December 25, 2020 (3). Human-to-human contact via droplets and aerosol has been linked to the transmission of the SARS-CoV-2 virus (4). On the 15 March 2020, the majority of countries all over the world started to impose a lockdown of their population and dental practices were asked to close temporarily. Not to mention, dentists were reported to be among the highest professional risk groups of being infected and a possible vector of the transmission of the SARS-CoV-2 virus (5).

Academic dental schools are considered as competitive and demanding learning communities. Although there have been many outstanding innovations in teaching methodologies during the last few years in higher education, the COVID-19 pandemic highlighted the structural difference between dental and medical schools in the methods of instruction, and the practical and clinical educational daily activities. In the academic dental schools, students have a 2-year preclinical and a 2-year clinical dental course curriculum designed to prepare them for licensure and practice upon graduation (6). Dental chairside teaching is far more complex than most higher education, as senior dental students take the responsibility for the management of the patient's oral health under guidance from clinical specialists (7). Moreover, clinical dental interventions demand the integration of both intellectual and technical skills, including a sensitivity to the patients' needs as well as technical ability, and most critical, risk consideration. In other words, dental students learn what should be done, when to intervene effectively, and finally how to critically appraise treatment outcomes.

All levels of the educational system in Egypt and worldwide have been affected by COVID-19 (8). The worldwide temporary closure of the academic institutions affected around 931,345,922 students in 94 countries representing 54% of total enrolled learners (9). Egyptian private and public universities either canceled or postponed all campus in-person events in order to minimize gatherings and hence control the routes of the virus transmission (10). However, these actions elevated the medical, economical, and social burden on both undergraduate and graduate communities (11). The experiences of oro-dental health education in clinical settings varied remarkably based on the national spread of the virus. Due to the rapid second wave spread of COVID-19, it is imperative for dental schools to apply dynamic adjustments to the delivery of educational clinical services and community services, in order to protect students, patients, and educators meanwhile sustaining continued student academic and research progress.

Some medical academic institutions have taken the action and documented their adjustments during the pandemic; yet; there is an urgent demand for further analysis and wide range screening of the condition in the Egyptian dental schools during the COVID-19 crisis. Thus, the current review aims to illustrate how one of public dental schools in Egypt acted in response to the COVID-19 pandemic and to present the ongoing challenges, and how academic and clinical educators have worked to overcome them.

## South Valley University Dental School Experience

On March 25, South Valley University undergraduate dental students were not allowed to perform in-person activities within the dental school and clinics based on the Egyptian prime minister decree for holding all the educational activities. Administrative and secretarial activities were only part-time accessible (2 days/week). Lectures and examinations moved on the web. The professors started recording video lectures to be available online on the e-learning platform of the University (*http://app.svu.edu.eg/ecourses/faculties3.aspx*). Moreover, intermediate and summative examinations have been performed online, except for final year students, who performed their written examination at the university halls following social distancing rules and strict protective infection control measures.

Early July, the university council regulated the process of carrying out clinical training for healthcare professionals. There were many specific challenges for educators which required rigorous attention in balancing the safety of dental students and staff and the adoption of effective protective measures whilst maintaining high quality educational services. The dental clinics remained available only for the management of dental emergencies. Restorative, esthetic, prophylactic, and all non-essential treatments were canceled. Mid October, the start of fall semester witnessed the restart of education activities on patients. Part of the clinical training activities of dental students was performed through case presentation, reading and critical appraisal of scientific literature, interactive discussions based on clinical scenarios, and update training in cross-infection control. The dental faculty administration placed strict rules to regulate the dental clinics including the limitation of patients' daily numbers, limitations of the accompanying person (one person allowed), temperature checking at the entrance of the clinics.

## Non-Clinical Education Challenges

According to the Egyptian Supreme Council of Universities regulations, the majority of Egyptian dental programs adopted the Hybrid Learning Model, in which lectures (theoretical part) were introduced via e-learning platforms, while in-person learning was limited to preclinical (laboratory), and practical (clinical) courses that cannot be delivered remotely. This strategy was implemented to minimize the physical interaction between students, in order to control the outspread of the infection. Meanwhile it allowed dental students to acquire their practical competencies essential for dental education and important for students' satisfaction, in spite of the compromised educational environment.

Teaching practical courses remotely requires resources and unhurried critical planning. For example, the presence of a stable internet connection is mandatory to provide online educational services, which might be a challenge to some of the developing countries. Besides, this new system requires a fundamental change in the mentality of both the faculty and students to perform differently in the educational process (10). Learning a new skill (preclinical or clinical) requires the integration of both; verbal and non-verbal educational methods (12). Dental students gain many practical skills without meaningful verbal expressions, such tacit knowledge usually provides a strong base to much of our dental skills (13). COVID-19 pandemic highlighted the need for educators and clinical teachers to understand the importance of tacit knowledge and how to provide alternative teaching modalities. Internationally, some dental programs started to monitor the students preclinical performance via novel methods like ZOOM, Microsoft Teams, and Whatsapp. The use of distant video illustrations, and the evaluation of the students' practical performance online might be a practical solution, although more studies are essential to evaluate their effectiveness. Some methods like the five-step method (14) can be modified for preclinical dental teaching. Before the students perform the procedure, the students visualize the procedure without any verbal explanation in order to help them to imagine the procedure before applying it. Then, the procedure demonstrated by the educator is repeated with verbal narration and detailed explanation of steps. Later, the students are asked to repeat the steps so that they can be internalized. And finally, the students perform the procedure with the educator observing and providing feedback as needed.

With the gradual breakthrough that has taken place in the lockdown procedures done in Egypt as well as many other countries, the majority of dental students have resumed their in-person practical classes since October 2020. The return to the practice had to deal with new challenges such as social distancing in classes, management of modified schedules, and the new mandatory public health guidelines (15). The Association for Dental Education, Asia Pacific (ADEAP) developed and published detailed guidelines for safe provision of dental education (including pre-clinical and clinical settings) during the COVID-19 pandemic (16). Although we serendipitously followed many of these items (like the majority of dental programs) during the pandemic (17), it's important to exchange these guidelines and distribute them globally in order to facilitate providing dental education in a safe environment. Regarding the in-person classes, dental academics need to emphasize strict infection control measures (protective face masks and shields, social distancing, and hand hygiene) among students, educators, and staff. The application of these measures is crucial for the safety of the work environment. Students, faculty, and staff should be only allowed to participate in limited, in-person classes, activities, and events that permit attendees to remain spaced at least six feet apart (e.g., lecture hall with seating spaced six feet apart). Besides, dental school should completely avoid out-of-class social activities and events and communications. It's important to continuously remind students, faculty, and staff not to share objects (e.g., laboratory and/or clinical supplies). Routinely scheduled cleaning and disinfection of frequently touched areas should be applied and monitored (e.g., on-time and consistently).

Despite these challenges, it may be a great opportunity to share experiences and information between dental schools, and to implement novel strategies in dental education, especially there are few hindrances to traditional dental learning scenarios. The main aim is to offer the dental students a new alternative that is satisfactory for their training. Although this might look enthusiastic, future studies are important to evaluate the impact of these guidelines and techniques on the learning outcomes, due to the unique demands of dental education (18).

## Practice-Based Clinical Challenges

The resumption of routine clinical dental activities was a difficult decision and should be guided by the virus updated status and national health guidelines to manage it. Dental academics had to plan for a safe recovery of clinical teaching for students, patients, educators, and staff with contingency plans. This is very crucial as SARS-CoV-2 is known to be highly contagious and the status of virus transmission in the Egyptian community remains unclear. Certain clinical training (like journal club and case presentation) were well-suited for teledentistry (19). In these cases, case presentation, and discussions could be done using online platforms (ZOOM, Webex, Microsoft Teams) (20). This approach can aid in conducting problem-focused interactions with students without the need of their physical presence (21, 22). On the other side, the therapeutic clinical training in the university clinics represented the main challenge for clinical dental education, specially the management of bio-aerosol, the insurance of the dental team compliance with the obligatory additional infection control measures, the application of social distancing policies to reduce overcrowding in the clinic, and thus reduce the exposure to others as well as the bio-aerosol generated during the clinical procedures (23).

### Dental Bio-Aerosols

Almost all dental procedures include a set of mechanical preparation, this includes the use of air turbines, air-water syringe, ultrasonic scalers, rotary handpieces, and air abrasion which generate bio-aerosols. Interestingly, these procedures include routine dental examination and hand-scaling which many dentists would consider safe to perform (24). Bio-aerosol generated during dental procedures contains a spectrum of microorganisms within tiny particles called droplet nuclei (1–5 μm) or droplets (>5 μm) (25). Large droplets tend to fall quickly due to the gravity, while droplet nuclei are airborne for hours due to their low settling velocity with the ability to propagate further before being inhaled inside the respiratory system or contaminate the clinic exposed surfaces (26).

Recent evidence demonstrated three main routes for the transmission of SARS-CoV-2 in dental settings: (1) direct transmission via inhalation of the virus via cough droplets; (2) transmission via oral, nasal, or eye membranes; and (3) direct contact transmission (27). All these three transmission routes are facilitated by dental procedures generated aerosols (28). It's important to highlight here that the majority of previous studies quantifying bio-aerosol generation during dental instrumentations have a range of differences in between them. Nevertheless, their findings should be taken into consideration when operating in this pandemic.

The reduction of bio-aerosol generation can be accomplished by using many protocols (26, 29). In our experience, we followed two different approaches to control bio-aerosol generation: (1) the use of pre-operative mouthwash; and (2) the regulation of daily dental procedures (30, 31). The use of pre-operative mouthwash (1% hydrogen peroxide, 0.2% povidone iodine, or alcohol free options such as 0.2% chlorhexidine rinse) has been shown to control the bacterial burden (23). In order to regulate the daily clinical practice, it was logical to prioritize senior dental students in the clinic as the most skillful clinical cohort with more structured clinical skills (32). Less experienced dental students have been found taking significantly longer time (2–3 h) than those of experienced dentists for doing the same clinical intervention, which exposes patients, students, educators, and staff to bio-aerosol for an extended period of time. Besides, it's preferable to prioritize patients with dental emergencies or acute dental pain, and mandate the use of the rubber dam (with aerosol generating procedures) until the situation becomes clearer or with the mass distribution of the vaccine.

### Infection Control

Infection control continues to be one of the most critical issues in healthcare service worldwide including dental schools. Infection control and prevention is crucial in providing a safe environment for students, patients, educators, and healthcare workers within clinical settings in dental schools (33–35). Since its establishment, the Faculty of Oral and Dental Medicine at South Valley University has followed the infection control regulations published in the Summary of Infection Prevention Practices in Dental Settings published by the Center for Disease Control and Prevention (CDC) as the standard infection control measures (36). The transmission of SARS-CoV-19 virus from human to human through the respiratory droplets of the patients (37) requires new regulations in order to control the infection outbreak.

Recently, multiple guidelines and recommendations have been published in order to share the modification in the infection control measures between dental professionals (26, 38). Although we applied many of these measures serendipitously, it is crucial to establish rational evidence-based protocols for all clinical settings to make sure that students, educators, and staff are understanding and applying these protocols properly, and thus do not compromise infection control measures especially at this crisis.

During the pandemic, we strictly applied the social-distancing regulation in the operating as well as patients' waiting rooms. Moreover, the clinic administrative board effectively monitored the hygiene levels in the clinic and patients waiting areas between appointments. This was achieved by using a hospital grade disinfectant that is effective against other viruses such as norovirus or 0.1% sodium hypochlorite or >60% alcohol based wipes (23). These applied measures are closely matching these applied in other countries like France, China, and Iran (15, 26, 38).

According to recent evidence, dental schools should perform primary screening when scheduling an appointment online or over the phone (26, 38). As we don't have online or phone scheduling at the dental clinics in South Valley University, screening of students, patients, faculty, and staff was applied including temperature checking and a questionnaire of any recent respiratory problems or fever at the entrance of the dental hospital. Inside the clinics, dental students, assistants, clinicians, and educators followed strictly the standard precautions for the airborne, as well as contact infections, which include but not limited the use of PPE and the hand hygiene protocols (39). Dental schools need to oblige students and assistants to use disposable surgical caps and long-sleeved polyethylene gowns with elasticized wrists which are cautiously disposed of after every patient. In some high-risk facilities, faculties, and/or clinical educators may be forced to change gowns between each student/patient, and patients are asked to wear disposable gowns. Furthermore, students, staff, and educators are required to wear a surgical mask or N95/P2 respirators inside the dental clinic. The N95/P2 surgical respirators have been known to provide more effective protection from both: airborne particles (0.3–0.6 μm) and from liquid droplets (40). Moreover, protective eyewear is an important protective gadget as the virus has been isolated from conjunctival samples from COVID-19 positive patients (41). Recent studies (42–45) showed that whilst dental students have a positive attitude toward infection control, their compliance is poor, specifically regarding eye protection, with 28% of senior students in some schools admitting to only use eyewear protection sometimes. Finally, all academic dental institutions should clinically assess the compliance of their students periodically to the infection control in the dental settings to ensure their understanding of the infection control obligations.

## Questions Concerning the Future of Dental Institutions and Education

Although COVID-19 pandemic has a worldwide unlimited devastating impact, this pandemic should alarm the health professionals to be more prepared for any future emerging pandemic. Dental academic institutions must learn from this experience and remember the binding professional responsibility and sharing information. Regarding dental clinical education, it's crucial to answer many questions that may help in understanding what could be done in the future; what will be the basic infection control standards, after the COVID-19 vaccination? The design and infrastructure of educational clinics including patients waiting areas may also need to be reconsidered in the future regarding the positioning, spacing of dental units, and the central air ventilation. In the same context, it is important to know when dental students and educators will receive the vaccine? Are they considered among high risk groups or just educational institutions? Institutional support plans for students, educators, and staff will be vital to help them dealing with the negative consequences of this current crisis.

Regarding pre-clinical dental education, the main challenge that e-learning faces is how to teach efficiently practical pre-clinical lessons in a safe environment. Since most of the dental subjects require practical interaction; therefore, it is not feasible to teach them only online. We believe that dental students will face a difficulty to fulfill the dental practical skills only with an online-based education system. That's why it is suggested that e-learning can be improved by enhancing its interactivity, showing dental procedures in real situations, in addition to providing 3D simulations to mimic the real teaching experience. Finally, It will be important to evaluate in the future the pedagogical effects of the structural change in the dental educational techniques caused by the COVID-19 crisis.

## Conclusion

The SARS-CoV-2 crisis has forced a paradigm shift on the performance of the dental academic institutions. Due to its uniqueness, clinical dental education faces special challenges compared to other medical specialties. Hybrid learning will probably be a cornerstone of future dental education. Educational clinical dental settings need to be re-adjusted as quickly as possible, according to the new infection control measures, to ensure high quality training for dental students, as well as continuation of community dental services, in a safe clinical environment. Finally, post-pandemic, dental academic institutions must consider documenting and sharing their guidelines and experiences in published research. This knowledge will help dental institutions to improve the overall educational process and to provide both pre-clinical and clinical dental education in a safe environment.

## Author Contributions

All authors have read and approved the final article. MGH conceived and designed the review. HA revised the manuscript. MGH and HA wrote and revised the final manuscript.

## Conflict of Interest

The authors declare that the research was conducted in the absence of any commercial or financial relationships that could be construed as a potential conflict of interest.

## References

[1] Li Q Guan X Wu P Wang X Zhou L Tong Y. Early transmission dynamics in Wuhan, China, of novel coronavirus-infected pneumonia. N Engl J Med. (2020) 382:1199–207. 10.1056/NEJMoa2001316 31995857 PMC7121484

[2] WHO/Europe. WHO Announces COVID-19 Outbreak a Pandemic. Available online at: https://www.euro.who.int/en/health-topics/health-emergencies/coronavirus-covid-19/news/news/2020/3/who-announces-covid-19-outbreak-a-pandemic (accessed December 25, 2020).

[3] WHO, Coronavirus Disease (COVID-19) Dashboard. Available online at: https://covid19.who.int (accessed December 25, 2020).

[4] Huang C Wang Y Li X Ren L Zhao J Hu Y. Clinical features of patients infected with 2019 novel coronavirus in Wuhan, China. Lancet. (2020) 395:497–506. 10.1016/S0140-6736(20)30183-5 31986264 PMC7159299

[5] Available, online at: https://www.osha.gov/Publications/OSHA3990.pdf (accessed December 30, 2020).

[6] Akinkugbe AA Garcia DT Smith CS Brickhouse TH Mosavel M. A descriptive pilot study of the immediate impacts of COVID-19 on dental and dental hygiene students' readiness and wellness. J Dent Educ. (2020) 85:401–10. 10.1002/jdd.12456 33084054 PMC8043566

[7] Sweet J Pugsley L Wilson J. Stakeholder perceptions of chairside teaching and learning in one UK dental school. Br Dent J. (2008) 205:499–503. 10.1038/sj.bdj.2008.934 18997709

[8] Nicola M Alsafi Z Sohrabi C Kerwan A Al-Jabir A Iosifidis C. The socio-economic implications of the coronavirus pandemic (COVID-19): a review. Int J Surg. (2020) 78:185–93. 10.1016/j.ijsu.2020.04.018 32305533 PMC7162753

[9] School Closures Caused by Coronavirus (Covid-19). Available online at: https://en.unesco.org/covid19/educationresponse (accessed December 26, 2020).

[10] Shehata MH Abouzeid E Wasfy NF Abdelaziz A Wells RL Ahmed SA. Medical education adaptations post COVID-19: an Egyptian reflection. J. Med. Educ. Curic. Dev. (2020) 7:2382120520951819. 10.1177/2382120520951819 32923673 PMC7457644

[11] Esposito S Principi N. School closure during the coronavirus disease 2019 (COVID-19) pandemic: an effective intervention at the global level? JAMA Pediatr. (2020) 174:921–2. 10.1001/jamapediatrics.2020.1892 32401277

[12] Maddox WT Ashby FG. Dissociating explicit and procedural-learning based systems of perceptual category learning. Behav Processes. (2004) 66:309–32. 10.1016/j.beproc.2004.03.011 15157979

[13] Fugill M. Tacit knowledge in dental clinical teaching. Eur J Dent Educ. (2012) 16:2–5. 10.1111/j.1600-0579.2011.00716.x 22251320

[14] George JH Doto FX. A simple five-step method for teaching clinical skills. Fam Med. (2001) 33:577–8. 11573712

[15] Gaudin A Arbab-Chirani R Pérez F. Effect of COVID-19 on dental education and practice in France. Front Dent Med. (2020) 1:5. 10.3389/fdmed.2020.00005

[16] Hong G Chang T-Y Terry A Chuenjitwongsa S Park Y-S Tsoi JK. Guidelines for innovation in dental education during the coronavirus disease 2019 pandemic. J Oral Sci. (2020) 63:107–10. 10.2334/josnusd.20-0399 33239486

[17] Gurgel BCdV Borges SB Borges REA Calderon PDS. COVID-19: perspectives for the management of dental care and education. J Appl Oral Sci. (2020) 28:e20200358. 10.1590/1678-7757-2020-0358 32997092 PMC7521424

[18] Ahmed SA Hegazy NN Malak HWA Kayser CW Elrafie NM Hassanien M. Model for utilizing distance learning post COVID-19 using (PACT)^TM^ a cross sectional qualitative study. BMC Med Educ. (2020) 20:400. 10.1186/s12909-020-02311-1 33138818 PMC7605338

[19] Farooq I Ali S Moheet IA AlHumaid J. COVID-19 outbreak, disruption of dental education, and the role of teledentistry. Pak J Med Sci Q. (2020) 36:1726–31. 10.12669/pjms.36.7.3125 33235605 PMC7674864

[20] Chang T-Y Hong G Paganelli C Phantumvanit P Chang W-J Shieh Y-S. Innovation of dental education during COVID-19 pandemic. J Dent Sci. (2021) 16:15–20. 10.1016/j.jds.2020.07.011 32839668 PMC7437532

[21] Abbas B Wajahat M Saleem Z Imran E Sajjad M Khurshid Z. Role of teledentistry in COVID-19 pandemic: a nationwide comparative analysis among dental professionals. Eur J Dent. (2020) 14:S116–22. 10.1055/s-0040-1722107 33383589 PMC7775233

[22] Yadav V Kumar V Sharma S Chawla A Logani A. Palliative dental care: ignored dimension of dentistry amidst COVID-19 pandemic. Spec Care Dentist. (2020) 40:613–5. 10.1111/scd.12517 32882066

[23] Sukumar S Dracopoulos SA Martin FE. Dental education in the time of SARS-CoV-2. Eur J Dent Educ. (2020) 25:325–31. 10.1111/eje.12608 33015929 PMC7675464

[24] Huntley DE Campbell J. Bacterial contamination of scrub jackets during dental hygiene procedures. J Dent Hyg. (1998) 72:19–23. 9693565

[25] Zemouri C de Soet H Crielaard W Laheij A. A scoping review on bio-aerosols in healthcare and the dental environment. PLoS ONE. (2017) 12:e0178007. 10.1371/journal.pone.0178007 28531183 PMC5439730

[26] Ge Z-Y Yang L-M Xia J-J Fu X-H Zhang Y-Z. Possible aerosol transmission of COVID-19 and special precautions in dentistry. J Zhejiang Univ Sci B. (2020) 21:361–8. 10.1631/jzus.B2010010 32425001 PMC7089481

[27] Pan Y Liu H Chu C Li X Liu S Lu S. Transmission routes of SARS-CoV-2 and protective measures in dental clinics during the COVID-19 pandemic. Am J Dent. (2020) 33:129–34. 32470237

[28] Innes N Johnson IG Al-Yaseen W Harris R Jones R Kc S. A systematic review of droplet and aerosol generation in dentistry. J Dent. (2021) 105:103556. 10.1016/j.jdent.2020.103556 33359043 PMC7834118

[29] Gandolfi MG Zamparini F Spinelli A Sambri V Prati C. Risks of aerosol contamination in dental procedures during the second wave of COVID-19-experience and proposals of innovative IPC in dental practice. Int J Environ Res Public Health. (2020) 17:8954. 10.3390/ijerph17238954 33271981 PMC7729834

[30] Bidra AS Pelletier JS Westover JB Frank S Brown SM Tessema B. Comparison of *in vitro* inactivation of SARS CoV-2 with hydrogen peroxide and povidone-iodine oral antiseptic rinses. J Prosthodont. (2020) 29:599–603. 10.1111/jopr.13220 32608097 PMC7361576

[31] Clementini M Raspini M Barbato L Bernardelli F Braga G Di Gioia C. Aerosol transmission for SARS-CoV-2 in the dental practice. A review by SIdP covid-19 task-force. Oral Dis. (2020). 10.1111/odi.13649. [Epub ahead of print]. 33124127

[32] Quinn B Field J Gorter R Akota I Manzanares M-C Paganelli C. COVID-19: the immediate response of european academic dental institutions and future implications for dental education. Eur J Dent Educ. (2020) 24:811–4. 10.1111/eje.12542 32394605 PMC7272881

[33] Bebermeyer RD Dickinson SK Thomas LP. Guidelines for infection control in dental health care settings–a review. Tex Dent J. (2005) 122:1022–6. 16350459

[34] Cleveland JL Bonito AJ Corley TJ Foster M Barker L Gordon Brown G. Advancing infection control in dental care settings: factors associated with dentists' implementation of guidelines from the Centers for Disease Control and Prevention. J Am Dent Assoc. (2012) 143:1127–38. 10.14219/jada.archive.2012.0044 23024311 PMC4624311

[35] Dagher J Sfeir C Abdallah A Majzoub Z. Infection control measures in private dental clinics in Lebanon. Int J Dent. (2017) 2017:5057248. 10.1155/2017/5057248 28642792 PMC5470049

[36] Centers for Disease Control and Prevention. Summary of Infection Prevention Practices in Dental Settings (2016).

[37] Wu Z Harrich D Li Z Hu D Li D. The unique features of SARS-CoV-2 transmission: comparison with SARS-CoV, MERS-CoV and 2009 H1N1 pandemic influenza virus. Rev Med Virol. (2020) 31:e2171. 10.1002/rmv.2171 33350025 PMC7537046

[38] Falahchai M Babaee Hemmati Y Hasanzade M. Dental care management during the COVID-19 outbreak. Spec Care Dentist. (2020) 40:539–48. 10.1111/scd.12523 32950037 PMC7537059

[39] Jadhav GR Mittal P. Coronavirus disease 2019: implications for clinical dental care. J Endod. (2020) 46:1341–2. 10.1016/j.joen.2020.05.026 32867925 PMC7455542

[40] Available, online at: http://www.cec.health.nsw.gov.au/__data/assets/pdf_file/0006/590307/Application-of-PPE-in-COVID-19.pdf (accessed December 26, 2020).

[41] Transmission of SARS-CoV-2: Implications for Infection Prevention Precautions. Available online at: https://www.who.int/news-room/commentaries/detail/transmission-of-sars-cov-2-implications-for-infection-prevention-precautions (accessed December 26, 2020).

[42] Ahmad IA Rehan EA Pani SC. Compliance of Saudi dental students with infection control guidelines. Int Dent J. (2013) 63:196–201. 10.1111/idj.12030 23879255 PMC9375015

[43] de Abreu MHNG Lopes-Terra MC Braz LF Rímulo AL Paiva SM Pordeus IA. Attitudes and behavior of dental students concerning infection control rules: a study with a10-year interval. Braz Dent J. (2009) 20:221–5. 10.1590/s0103-64402009000300009 19784468

[44] Acosta-Gío AE Borges-Yáñez SA Flores M Herrera A Jerónimo J Martínez M. Infection control attitudes and perceptions among dental students in Latin America: implications for dental education. Int Dent J. (2008) 58:187–93. 10.1111/j.1875-595x.2008.tb00347.x 18783110

[45] Hoover TE Kirk J Fredekind RE. Clinical quality assurance surveillance and targeted interventions: managing unfavorable trends in a dental school clinic. J Dent Educ. (2007) 71:746–58. 10.1002/j.0022-0337.2007.71.6.tb04331.x 17554092

